# Different Flour Microbial Communities Drive to Sourdoughs Characterized by Diverse Bacterial Strains and Free Amino Acid Profiles

**DOI:** 10.3389/fmicb.2016.01770

**Published:** 2016-11-08

**Authors:** Giuseppe Celano, Maria De Angelis, Fabio Minervini, Marco Gobbetti

**Affiliations:** Dipartimento di Scienze del Suolo, della Pianta e degli Alimenti, Università degli Studi di Bari Aldo MoroBari, Italy

**Keywords:** irradiated flour, lactic acid bacteria, yeasts, enterobacteria, sourdough, bacterial strains, free amino acids

## Abstract

This work aimed to investigate whether different microbial assemblies in flour may influence the microbiological and biochemical characteristics of traditional sourdough. To reach this purpose, members of lactic acid bacteria, enterobacteria, and yeasts were isolated from durum wheat flour. Secondly, the isolated microorganisms (*Pediococcus pentosaceus, Saccharomyces cerevisiae, Pantoea agglomerans*, and *Escherichia hermannii*) were inoculated in doughs prepared with irradiated flour (gamma rays at 10 kGy), so that eight different microbial assemblies were obtained. Two non-inoculated controls were prepared, one of which (C-IF) using irradiated flour and the other (C) using non-irradiated flour. As shown by plate counts, irradiation of flour caused total inactivation of yeasts and a decrease of all the other microbial populations. However, acidification occurred also in the dough C-IF, due to metabolic activity of *P. pentosaceus* that had survived irradiation. After six fermentations, *P. pentosaceus* was the dominant lactic acid bacterium species in all the sourdoughs produced with irradiated flour (IF). Yet, IF-based sourdoughs broadly differed from each other in terms of strains of *P. pentosaceus*, probably due to the different microorganisms initially inoculated. Quantitative and qualitative differences of free amino acids concentration were found among the sourdoughs, possibly because of different microbial communities. In addition, as shown by culture-independent analysis (16S metagenetics), irradiation of flour lowered and modified microbial diversity of sourdough ecosystem.

## Introduction

Sourdough positively influences the sensory, nutritional, textural, and shelf-life features of leavened baked goods (De Vuyst et al., 2009; Gobbetti et al., 2014). This biotechnological method allows artisanal and industrial bakeries to respond to the increasing consumers' demand for baked goods having higher overall quality than those obtained with baker's yeast (Catzeddu, 2011).

Sourdough originates from the spontaneous or starter culture-initiated fermentation of mixtures of flour and water (Hammes and Gänzle, 1997). Compared to industrial, traditional sourdoughs are characterized by larger microbial diversity (De Vuyst et al., 2009), mainly because spontaneous multi-step fermentation is needed for their preparation (Hammes and Gänzle, 1997). In the first step a dough, usually composed of just flour and water, is spontaneously fermented. At this stage, redox potential decreases (Hammes et al., 2005), favoring the growth of facultatively anaerobes (*Enterobacteriaceae* and yeasts) and lactic acid bacteria (LAB). After fermentation, the dough is used as inoculum for fermenting newly prepared dough, which, in turn, will be used as inoculum for a subsequent step of fermentation (Minervini et al., 2014). During sourdough preparation, Gram-positive bacteria usually outgrow Gram-negative bacteria (Onno and Roussel, 1994). Within Gram-positive bacteria, a microbial succession involving LAB is well-known. In detail, from initial dominance of coccus-shaped LAB, the microbial population in most of cases becomes represented by lactobacilli (Van der Meulen et al., 2007; Weckx et al., 2010). This is because lactobacilli better adapt to the low pH distinctive of sourdough and, more in general, to other characteristic conditions of this ecosystem, such as time and temperature of fermentation and concentration of oxygen (Mihhalevski et al., 2011). Microbial community of mature sourdough includes LAB and yeasts. However, *Enterobacteriaceae* (Scheirlinck et al., 2008; Gu et al., 2014) and acetic acid bacteria (Scheirlinck et al., 2008; Vogelmann et al., 2009; Minervini et al., 2012a) may be rarely detected in the mature sourdough.

Traditional sourdoughs may differ from each other in terms of microbial diversity, which is driven by: (i) specific technology parameters; (ii) house microbiota; and (iii) flour (Minervini et al., 2014, 2015). Flour affects sourdough microbiota because of its content in nutrients and contaminating microorganisms, mainly bacteria belonging to *Proteobacteria* (e.g., *Enterobacter* sp., *Pantoea* sp., and *Pseudomonas* sp.) and *Firmicutes* (e.g., *Lactobacillus* sp., *Leuconostoc* sp., and *Weissella* sp.) phyla (Ercolini et al., 2013; De Vuyst et al., 2014). Like all the spontaneously fermented food, sourdough fermentation may fail. Dominance of *Proteobacteria* during preparation of sourdough could be one of the causes of failure. That is why some sourdough producers use additional ingredients in early fermentation steps (Minervini et al., 2016). Although microbial ecology dynamics characterizing sourdough preparation were previously clarified (Van der Meulen et al., 2007; Weckx et al., 2010; Ercolini et al., 2013), to our knowledge, so far no study tried to understand how autochthonous flour microorganisms affect the characteristics of traditional sourdough.

This work aimed to investigate whether different microbial assemblies in flour may influence the microbiological and biochemical characteristics of sourdough. To reach this purpose, members of LAB, enterobacteria, and yeasts were isolated from durum wheat flour. Secondly, the isolated microorganisms were inoculated in doughs prepared with flour treated with gamma rays. Thereafter, doughs were propagated, under laboratory conditions, for 6 days. The obtained sourdoughs were studied by a multi-phasic approach.

## Materials and methods

### Treatment of durum wheat flour

Commercial durum wheat flour was kindly provided by L'Antico Molino Calemma (Altamura, Bari, Italy). The gross composition was as follows: Moisture, 14.9%; protein (N × 5.7), 12.2%; total carbohydrates, 71.1% (maltose, 0.70%; glucose, 0.34%; fructose, 0.40%); fat, 1.7%. Flour (5 kg) in paper bags was placed in a cardboard box and exposed to 60Co γ-ray source at Gammatom s.r.l. (Guanzate, Como, Italy). Samples were irradiated with a dose of 10 kGy. Non-irradiated flour was used as the control.

### Alpha-amylase activity

The alpha-amylase activity of the flour, before and after irradiation, was estimated through determination of the falling number. Falling number was determined in triplicate according to AACCI method 56-81.03 (AACC, 2000).

### Microbiological analyses

Ten grams of flour were homogenized with 90 or 20 ml of sterile peptone water (peptone 1 g l^−1^ and NaCl 8.5 g l^−1^) solution (Minervini et al., 2015), before and after irradiation, respectively. Total mesophilic aerobic microorganisms, presumptive LAB, enterococci, staphylococci, enterobacteria, “flat-sour” bacteria, *Pseudomonas* sp., acetic acid bacteria, and yeasts were enumerated using the agar media reported in Table 1 (Minervini et al., 2015). In addition, serial dilutions of irradiated flour (IF) were maintained in a water bath at 80°C for 15 min to activate spores, plated in Plate Count agar and incubated at 30°C (under aerobic or anaerobic conditions) for up to 76 h (Aziz et al., 2006).

**Table 1:** **Culture media, method of inoculum, time (h) and temperature (°C) of incubation used for enumerating different microbial groups**.

**Microbial group**	**Medium for enumeration^a^**	**Method of inoculum**	**Time of incubation (h)**	**Temp of incubation (°C)**
Total mesophilic aerobic microorganisms	PCA	Pour-plate	48	30
Lactic acid bacteria	MRS with cycloheximide (0.1 g l^−1^)	Pour-plate	48	30
	SDB with cycloheximide (0.1 g l^−1^)	Pour-plate	48	30
Enterococci	Slanetz and Bartley	Pour-plate	48	37
Staphylococci	Baird Parker	Spread-plate	24	37
“Flat-sour” bacteria	Dextrose tryptone	Pour-plate	72	30
Enterobacteria	VRBG	Pour-plate	24	37
*Pseudomonas* sp.	*Pseudomonas* agar base with cetrimide (0.01 g l^−1^), fucidin (0.01 g l^−1^), cephaloridine (0.05 g l^−1^)	Spread-plate	24	30
Acetic acid bacteria	Glucose solid GYC with cycloheximide (0.1 g l^−1^)	Spread-plate	96	30
Yeasts	Sabouraud Dextrose with chloramphenicol (0.1 g l^−1^)	Pour-plate	48	30

a *All the culture media were produced by Oxoid Ltd. (Basingstoke, UK), with the exceptions of SDB and Glucose solid GYC, which were laboratory-made*.

### Isolation and identification of bacteria and yeasts from non-irradiated flour

Fifteen colonies of presumptive LAB were picked up from the (MRS/SDB) plates containing the highest dilutions of flour, transferred into the corresponding broth media and re-streaked until pure cultures were obtained.

Five colonies of presumptive yeasts were picked up from the Sabouraud Dextrose agar plate containing the highest dilutions of flour, sub-cultured in the corresponding broth medium and re-streaked onto the same agar medium. All the isolates could ferment galactose, sucrose, maltose, and raffinose and could grow at 37°C.

Twenty colonies of presumptive *Enterobacteriaceae* were picked up from the VRBGA plate containing the highest dilutions of flour, sub-cultured in Tryptone Soy broth and re-streaked onto Tryptone Soy agar.

Bacterial and yeast isolates were identified by partial sequencing of the *16S rRNA* and *26S rRNA* genes, respectively. In detail, genomic DNA was extracted from bacteria using DNeasy Blood and Tissue Kit (Qiagen, SA, Courtaboeuf, France), according to the manufacturer's instructions (Ahmed et al., 2009). The DNA was used as template in PCR using primers LacbF/LacbR (Corsetti et al., 2004). DNA was extracted from yeasts using the Wizard Genomic DNA Purification kit (Promega Corporation, Madison, WI), according to the manufacturer's instructions (Soteropoulos and Perlin, 1998). The DNA was used as template in PCR using primers NL-1/NL-4, targeting the D1/D2 domain of the *26S rRNA* gene (Kurtzman and Robnett, 1998). After purification with GFX PCR DNA gel band purification kit (GE Healthcare Bio-Sciences), PCR products of LAB, *Enterobacteriaceae* and yeasts were sequenced at Eurofins Genomics (Ebersberg, Germany). Pair-wise sequence alignments were carried out using the BLAST search in the NCBI Nucleotide Collection Database (Altschul et al., 1990).

### Sourdough preparation

Ten sourdoughs were prepared according to traditional protocols (Minervini et al., 2012b). Flour (120 g) and sterile tap water (60 g) were kneaded with a continuous high-speed mixer (60 × g, dough mixing time 5 min) (Chopin & Co., Boulogne, Seine, France). In order to obtain artificially assembled microbial communities, eight doughs (dough yield 160) were prepared with IF and initially inoculated with different combinations (Table 2) of the following microorganisms isolated from non-irradiated flour: *Pediococcus pentosaceus* A1, *Saccharomyces cerevisiae* SDA1, *Pantoea agglomerans* DTB8, and *Escherichia hermannii* PS2. All the possible combinations were tested, with the exception of those excluding *P. pentosaceus*. Liquid cultures (24–48 h) of the above strains were centrifuged (4528 × g for 5 min at 4°C). The harvested cells were washed twice with sterile saline (NaCl 9 g l^−1^) solution and singly re-suspended, to a final cell density in the range 2–3 log cfu g^−1^, in the tap water used for preparing the initial dough (Lattanzi et al., 2014). Before and after each kneading step, the mixer surfaces contacting the dough were washed, wiped with ethanol, and rinsed with sterile water. Two not inoculated controls were prepared, one of which using IF (C-IF) and the other using non-irradiated flour (C).

**Table 2:** **Microorganisms isolated from durum wheat flour before irradiation and used to inoculate initial doughs prepared with irradiated (γ-rays at 10 kGy) flour (IF)**.

**Dough**	**Strains**
D1-IF	*Pediococcus pentosaceus* A1^c^ (2 log cfu g^−1^)
D2-IF	*P. pentosaceus* A1 (2 log cfu g^−1^), *Saccharomyces cerevisiae* SDA1^d^ (2 log cfu g^−1^)
D3-IF	*P. pentosaceus* A1 (2 log cfu g^−1^), *Pantoea agglomerans* DTB8^e^ (3 log cfu g^−1^)
D4-IF	*P. pentosaceus* A1 (2 log cfu g^−1^), *S. cerevisiae* SDA1 (2 log cfu g^−1^), *Pa. agglomerans* DTB8 (3 log cfu g^−1^)
D5-IF	*P. pentosaceus* A1 (2 log cfu g^−1^), *Escherichia hermannii* PS2^f^ (3 log cfu g^−1^)
D6-IF	*P. pentosaceus* A1 (2 log cfu g^−1^), *S. cerevisiae* SDA1 (2 log cfu g^−1^), *E. hermannii* PS2 (3 log cfu g^−1^)
D7-IF	*P. pentosaceus* A1 (2 log cfu g^−1^), *Pa. agglomerans* DTB8 (3 log cfu g^−1^), *E. hermannii* PS2 (3 log cfu g^−1^)
D8-IF	*P. pentosaceus* A1 (2 log cfu g^−1^), *S. cerevisiae* SDA1 (2 log cfu g^−1^), *Pa. agglomerans* DTB8 (3 log cfu g^−1^), *E. hermannii* PS2 (3 log cfu g^−1^)
C-IF^a^	–
C^b^	–

*The initial cell density (log cfu g^−1^) of each strain is indicated in brackets. Control doughs were prepared without inoculation by using IF (C-IF) and non-irradiated durum wheat flour (C)*.

a *Dough prepared with irradiated flour, without inoculation*.

b *Dough prepared with non-irradiated flour, without inoculation*.

c *Isolated from MRS agar plate inoculated with a 10^−2^ diluted suspension of non-irradiated flour*.

d *Isolated from Sabouraud Dextrose agar plate inoculated with a 10^−2^ diluted suspension of non-irradiated flour*.

e *Isolated from VRBGA plate inoculated with a 10^−3^ diluted suspension of non-irradiated flour*.

f *Isolated from VRBGA plate inoculated with a 10^−3^ diluted suspension of non-irradiated flour*.

Daily, each sourdough was subjected to fermentation at 30°C for 6 h. The only exception was the first fermentation, which lasted 8 h according to traditional protocols (Minervini et al., 2012b). Aliquots (10 g) of the C and C-IF doughs were collected after the first fermentation, stored (at −80°C) in 10 ml of RNAlater® diluted (1:1) with water, and analyzed by culture-independent method (16S metagenetics). After each fermentation, sourdoughs were stored at 4°C for ca. 16 h. Then, each sourdough was propagated for 5 consecutive days, by one daily back-slopping step, using 90 g of flour (IF in all the cases, except for the C dough), 45 g of fermented dough, and 45 g of sterile tap water. Each type of sourdough was produced in triplicate. Dough volume and pH were determined at the beginning and end of fermentation. ΔpH was calculated as the difference between the values of pH at the beginning and end of fermentation (Lattanzi et al., 2014). Mature sourdoughs, obtained after five back-slopping steps, were further analyzed.

### Determination of carbohydrates, organic acids, ethanol, and free amino acids

Ten grams of mature sourdough were homogenized with 90 ml of Tris-HCl 50 mM pH 8.8 buffer and treated for 3 min in a Bag Mixer 400P (Interscience, St Nom, France) blender. After incubation (at 25°C for 30 min under stirring), the water-soluble extract was obtained by centrifugation (12,857 × g, 10 min, 4°C). Maltose, glucose, fructose, lactic acid, acetic acid, and ethanol were determined in the water-soluble extract of sourdoughs by High Performance Liquid Chromatography (HPLC) (Zeppa et al., 2001), using an ÄKTA Purifier™ system (GE Healthcare Bio-Sciences, Uppsala, Sweden) equipped with a 300 mm × 7.8 mm i.d. cation exchange column (Aminex HPX-87H, Bio-Rad Laboratories, CA) and a refractive index detector (Perkin Elmer Corp., Waltham, MA). The concentration of free amino acids (FAA) in the water-soluble extract of sourdoughs was determined using the Biochrom 30 Amino Acid Analyser (Biochrom LTD, Cambridge Science Park, England) as previously described (De Angelis et al., 2007).

### Lactic acid bacteria, enterobacteria and yeasts enumeration, and isolation

LAB, enterobacteria and yeasts were enumerated in sourdoughs after each fermentation step. Before inoculating the media, 10 g of sourdoughs were homogenized with 90 ml of sterile peptone water as previously described (Minervini et al., 2012b). For each mature sourdough batch, at least ten colonies of presumptive LAB and yeasts were randomly selected from the plates containing the highest sample dilutions, provided that the number of colonies on plates ranged from 100 to 300. Pure microbial cultures were obtained as described above.

### Genotypic characterization and identification of lactic acid bacteria and yeasts

Genomic DNA of LAB and yeasts was extracted as described above. Biotyping of LAB was carried out through Random Amplification of Polymorphic DNA (RAPD)-PCR using primers P4, P7 (De Angelis et al., 2001), and M13 (Siragusa et al., 2009). Yeasts were biotyped through RAPD-PCR using primers M13m and RP11 (Del Bove et al., 2009). RAPD-PCR profiles were acquired by the microchip electrophoresis system MCE-202 MultiNA (Shimadzu Italia s.r.l., Milano, Italy), as previously described (Minervini et al., 2016). The similarity of the electrophoretic profiles was evaluated by the Pearson product moment correlation coefficient (r) and using the Unweighted Paired Group Mathematic Average (UPGMA) algorithm. Identification of bacterial and yeast strains was performed as described above.

### Total bacterial RNA extraction and analysis of bacterial diversity

Ninety milliliters of saline solution were added to 10 g of dough and homogenized for 5 min, and RNA was extracted from doughs produced in three different fermentation trials performed in different days (Minervini et al., 2015). The purified RNA was reverse-transcribed according to the method reported by Gowen and Fong (2010). cDNA from doughs produced in three fermentation trials was pooled, dried using a vacuum centrifuge (SpeedVac Concentrator SPD121P, Thermo Scientific) and used as template for 16S metagenetics, which were carried out at the Research and Testing Laboratory (2016) (RTL, Lubbock, TX), by using the Illumina MiSeq platform. A fragment of the *16S rRNA* gene for analysis of diversity inside the domain of *Bacteria* was amplified using the primers 28F (GAGTTTGATCNTGGCTCAG) (Handl et al., 2011) and 388R (TGCTGCCTCCCGTAGGAGT) (Francés et al., 2004). PCR and sequencing analyses were carried out according to the protocol of RTL.

The sequenced reads were processed through denoising and chimera detection. In details, denoising was performed through the following steps: (i) merging together the forward and reverse reads using the PEAR Illumina paired-end read merger (Zhang et al., 2014); (ii) grouping reads (having an average quality higher than 25) by using the USEARCH (Edgar, 2010) algorithm (prefix dereplication) into clusters (4% dissimilarity among sequences of the same cluster), so that each sequence of shorter length to the centroid sequence must be a 100% match to the centroid sequence for the length of the sequence; (iv) Operational Taxonomic Unit (OTU) selection by using the UPARSE OTU selection algorithm (Edgar, 2013). Following denoising, the selected OTU were chimera checked using the UCHIME software (Edgar et al., 2011). In detail, each trimmed read was mapped to its corresponding non-chimeric cluster using the USEARCH global alignment algorithm (Edgar, 2010). Each sequence in a cluster was then aligned to the consensus sequence. Each sequence was corrected base by base in order to remove noise. Analysis of microbial diversity was finally performed by running the centroid sequence from each cluster against the USEARCH algorithm, using a database of high quality sequences derived from the NCBI. Lastly, the output was analyzed using an internally developed python program that assigns taxonomic information to each sequence.

The percentage of each bacterial OTU was analyzed individually for each sample, providing relative abundance information among the samples based on the relative numbers of reads within each (Andreotti et al., 2011). Alpha diversity (Chao 1 richness and Shannon diversity indices) was calculated using QIIME (Shannon and Weaver, 1949; Chao and Bunge, 2002; Suchodolski et al., 2012).

### Statistical analyses

Data (at least three replicates) of pH, lactic acid, acetic acid, ethanol, carbohydrates, FAA, and cell density of presumptive LAB and yeasts were subjected to one-way ANOVA, and pair-comparison of treatment means was achieved by Tukey's procedure at *P* <0.05, using a statistical software (Statistica 7.0 per Windows). Principal Component Analysis (PCA) was also performed using Statistica 7.0.

### Nucleotide sequence accession number

The sequence data were submitted to the sequence read archive of NCBI database and the corresponding accession no. is PRJNA318402.

## Results

### Effects of irradiation on microbial community and alpha-amylase activity of flour

**Figure 1:**
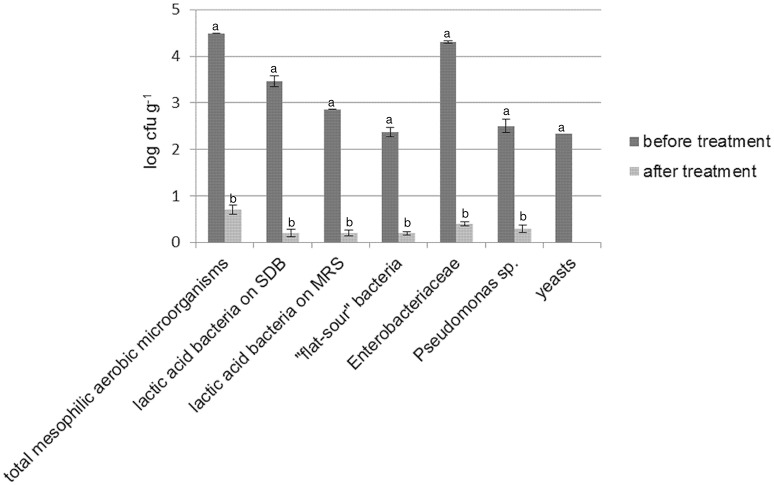
**Cell density (log cfu g^**−1**^) of total mesophilic aerobic microorganisms, presumptive lactic acid bacteria (enumerated on both SDB and MRS), “flat-sour” bacteria, ***Enterobacteriaceae***, ***Pseudomonas*** sp., and yeasts in durum wheat flour before and after treatment with γ-rays at 10 kGy**. Within the same microbial group, histogram bars with different letter are significantly different (*P* <0.05).

As shown by plate counts, durum wheat flour was contaminated especially by presumptive *Enterobacteriaceae* (Figure 1). LAB, “flat-sour” bacteria (e.g., *Bacillus* sp.), *Pseudomonas* sp. and yeasts were found at lower cell density. Enterococci, staphylococci and acetic acid bacteria were not detected. Irradiation of flour resulted in a reduction (from two to four log cycles) of all the microbial populations. Overall, bacteria were found at cell density lower than 1 log cfu g^−1^, whereas yeasts were not detected in 10 g of flour. No colonies (presumptive spore-formers) were detected in PCA plates inoculated with irradiated flour (IF) serially diluted and subjected to heat treatment (data not shown). The alpha-amylase activity of IF was significantly higher than flour prior to irradiation (272 ± 10 vs. 367 ± 15 s).

### Acidification and leavening during production of sourdoughs

IF was used to prepare and propagate doughs inoculated (D1-IF, D2-IF, D3-IF, D4-IF, D5-IF, D6-IF, D7-IF, D8-IF) or not (C-IF) with one or more microorganisms isolated from flour before irradiation. The different microbial combinations for inoculating doughs were chosen on the basis of the dominating microorganisms isolated from non-irradiated flour. A control sourdough (C) was prepared by using non-irradiated flour in both the first fermentation and the consecutive back-slopping steps. Compared to C-IF and C sourdoughs, dough acidification was faster in all the inoculated doughs already after the first back-slopping (Supplementary Table 1). Overall, mean values of ΔpH tended to stabilize after the last back-slopping or even earlier (D1-IF, D2-IF, D3-IF, D4-IF).

Slight volume increase (2–5 ml) was found after the first back-slopping only for doughs D6-IF, D7-IF, D8-IF, and C doughs (Supplementary Table 1). Except for D2-IF dough, no doughs showed any volume increase between the second and the third back-slopping steps. After the fourth back-slopping, D6-IF, D8-IF and, especially, D2-IF increased their volume. After the fifth back-slopping, these doughs showed volume increase, along with D4-IF, ranging from 10 to 20 ml. On the contrary, all the other sourdoughs (included the control) did not leaven.

### Biochemical and microbiological characteristics of sourdoughs

During propagation of sourdoughs, cell densities of LAB increased in all the doughs (Supplementary Table 2). *Enterobacteriaceae* decreased and, in most of cases, were not detected already after the fermentation following the second back-slopping step. Yeasts increased in the D2-IF, D4-IF, D6-IF, D7-IF, and D8-IF doughs. The cell density of LAB in the mature sourdoughs, as estimated on SDB, ranged from ca. 8.8 (D1-IF sourdough) to ca. 9.3 (D7-IF and C sourdoughs) log cfu g^−1^ (*P* > 0.05) (Supplementary Figure 1). Compared to SDB, no significant differences (*P* > 0.05) were found when cell density of LAB was estimated on MRS. Cell density of yeasts was below 1.0 log cfu g^−1^ for the D1-IF, D3-IF, D5-IF, C-IF, and C sourdoughs. For the remaining sourdoughs, this parameter ranged from ca. 2.8 (D7-IF) to ca. 7.4 (D2-IF) log cfu g^−1^.

Mature sourdoughs showed pH values ranging from ca. 4.00 (C sourdough) to ca. 4.50 (C-IF sourdough) (Table 3). The concentration of lactic acid was in agreement with the pH and ranged from ca. 48 to ca. 58 mM. Acetic acid was below the limit of detection (0.05 mM) in all the sourdoughs, except for D1-1F. Ethanol varied from ca. 6 (C) to ca. 463 (D2-IF) mM. The range of FAA was from ca. 340 (D2-IF) to ca. 1440 (C) mg kg^−1^. Among individual FAA, asp, thr, ile, leu, trp, and γ-aminobutyric acid (GABA) showed the largest variation (Supplementary Table 3). Residual maltose varied from ca. 0.31% (D2-IF) to 3.14% (C-IF) and glucose from ca. 0.05% (D6-IF) to 0.15% (D1-IF and D7-IF) (Table 3). Residual fructose ranged from ca. 0.14 to 0.21%.

**Table 3:** **pH values, concentrations of lactic acid (millimoles kg^**−1**^), acetic acid (millimoles kg^**−1**^), ethanol (millimoles kg^**−1**^), total free amino acids (FAA, in mg kg^**−1**^), maltose (%, g/100 g), glucose (%, g/100 g), and fructose (%, g/100 g) of mature sourdoughs prepared with irradiated durum wheat flour (IF) or non-irradiated flour (C)**.^a^

**Sourdough**	**pH**	**Lactic acid**	**Acetic acid**	**Ethanol**	**FAA**	**Maltose**	**Glucose**	**Fructose**
D1-IF	4.05 (0.01)^c^	54 (3)^bc^	1 (0)^a^	40 (2)^e^	1175 (20)^b^	3.03 (0.04)^bc^	0.15 (0.01)^a^	0.19 (0.00)^a^^b^
D2-IF	4.12 (0.03)^b^	51 (1)^cd^	< 0.05	463 (10)^a^	340 (14)^h^	0.31 (0.02)^g^	0.08 (0.02)^bc^	0.14 (0.02)^c^
D3-IF	4.06 (0.00)^c^	55 (5)^a^^b^	< 0.05	40 (3)^e^	1070 (23)^c^	3.12 (0.04)^a^^b^	0.14 (0.02)^a^	0.18 (0.02)^a^^b^
D4-IF	4.07 (0.01)^c^	56 (5)^a^^b^	< 0.05	153 (8)^b^	745 (19)^e^	2.46 (0.00)^e^	0.08 (0.00)^bc^	0.21 (0.01)^a^
D5-IF	4.05 (0.02)^c^	55 (2)^a^^b^	< 0.05	24 (1)^f^	920 (15)^d^	2.84 (0.02)^d^	0.12 (0.01)^a^^b^	0.15 (000)^bc^
D6-IF	4.04 (0.02)^cd^	57 (1)^a^^b^	< 0.05	112 (5)^d^	610 (11)^f^	1.95 (0.01)^f^	0.05 (0.02)^c^	0.14 (0.01)^c^
D7-IF	4.04 (0.00)^cd^	58 (4)^a^	< 0.05	24 (1)^f^	920 (15)^d^	3.11 (0.03)^a^^b^	0.15 (0.03)^a^	0.17 (0.01)^bc^
D8-IF	4.07 (0.03)^c^	55 (3)^a^^b^	< 0.05	137 (8)^c^	585 (16)^f^	1.89 (0.02)^f^	0.06 (0.01)^c^	0.15 (0.02)^bc^
C-IF	4.50 (0.02)^a^	48 (3)^d^	< 0.05	< 0.05	545 (15)^g^	3.14 (0.05)^a^	0.12 (0.03)^a^^b^	0.17 (0.03)^bc^
C	4.00 (0.02)^d^	58 (2)^a^	< 0.05	6 (1)^g^	1440 (19)^a^	2.93 (0.02)^cd^	0.09 (0.01)^bc^	0.14 (0.02)^c^

a *Values are means (three biological replicates, two analytical replicates). Values within a column with different superscript letters are significantly different (P <0.05)*.

### Characterization and identification of lactic acid bacteria and yeasts in sourdoughs

**Figure 2:**
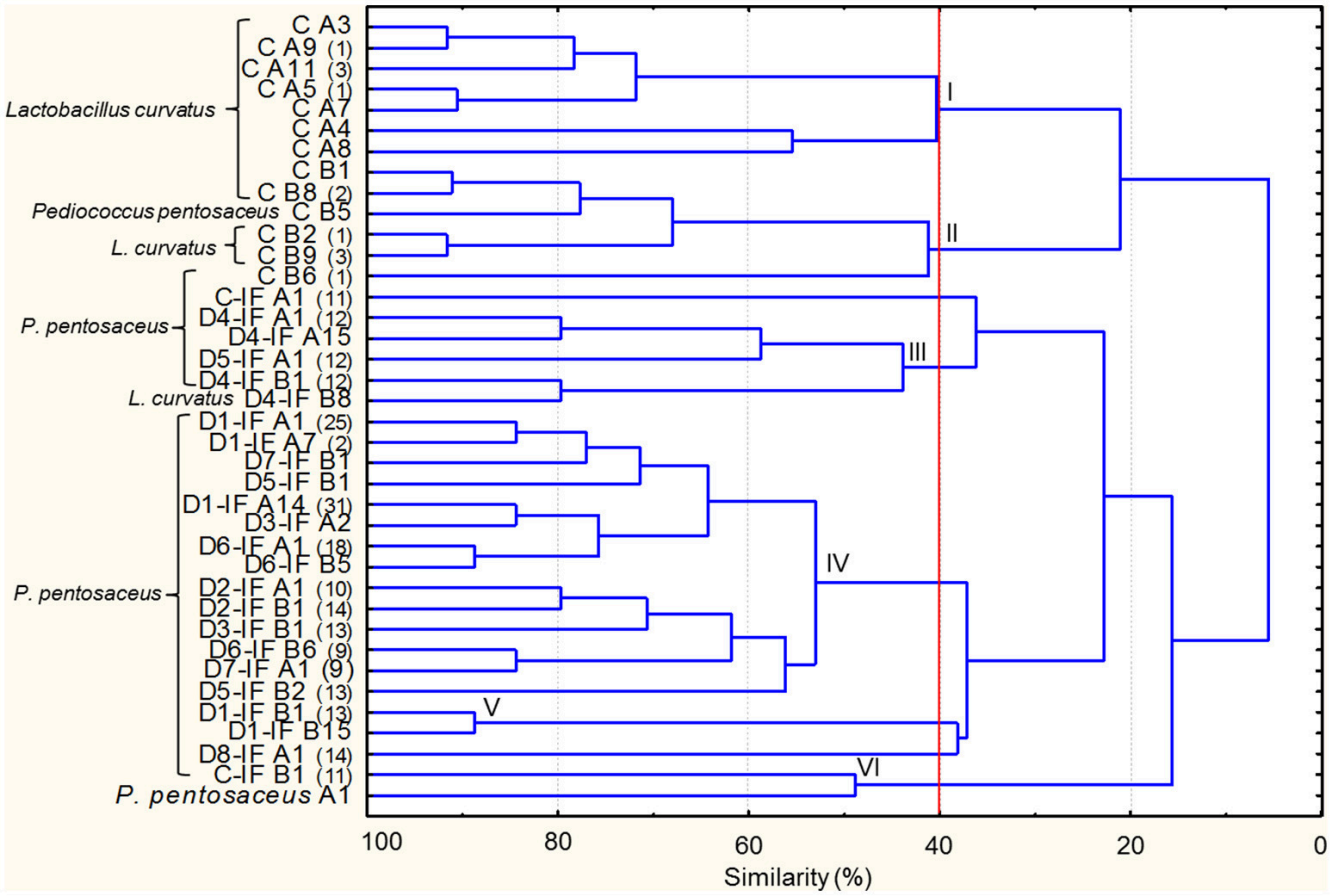
**Dendrogram of combined (primers P4, P7, and M13) RAPD profiles of lactic acid bacterium strains isolated from the mature sourdoughs prepared with irradiated durum wheat flour (IF) or non-irradiated flour (C)**. The first letters (C or C-IF) or alphanumeric code (D1-IF, D2-IF, D3-IF, D4-IF, D5-IF, D6-IF, D7-IF, D8-IF) of the strain name indicates the sourdough of origin, whereas the following letter (separated by one space) of the strain name indicates the medium used for the isolation (A, mMRS; B, SDB). Where present, the numbers in brackets at right of the strain name indicates the number of isolates showing the same RAPD profile. *Pediococcus pentosaceus* A1 isolated from flour has been included in the dendrogram. Cluster analysis was based on UPGMA algorithm. Clusters are indicated by Roman numerals (I–VI).

Microbial cultures isolated from mature sourdoughs are listed in Supplementary Table 4. Two-hundred-thirty-two Gram-positive, catalase-negative, non-motile, cocci, and rods acidifying isolates were subjected to RAPD analysis resulting in 37 strains (Figure 2). The inoculated strain (*P. pentosaceus* A1) was included in the dendrogram, showing that no *P. pentosaceus* isolated from sourdoughs showed the same profile as A1. At 40% of similarity, strains were grouped in six clusters (I–VI), with two unclustered. Clusters I and II exclusively grouped all the strains isolated from the sourdough produced by using non-irradiated flour (C). The largest cluster (IV) grouped strains isolated from different sourdoughs produced by using IF. The C sourdough was characterized by the highest number of strains (13), whereas the others harbored from one to five strains. *P. pentosaceus* was identified in all the mature sourdoughs. *Lactobacillus curvatus* was found as dominant and sub-dominant species in the C and D4-IF sourdoughs, respectively (Figure 2 and Supplementary Table 5). *P. pentosaceus* D1-IF A1 showed the same RAPD profiles as four and eleven isolates from the sourdoughs D2-IF and D7-IF, respectively (Supplementary Table 5). Likewise, another strain of *P. pentosaceus* isolated from the D1-IF sourdough (D1-IF A14) shared the same profiles as thirteen, three, and fifteen isolates from the sourdoughs D3-IF, D7-IF, and D8-IF, respectively.

**Figure 3:**
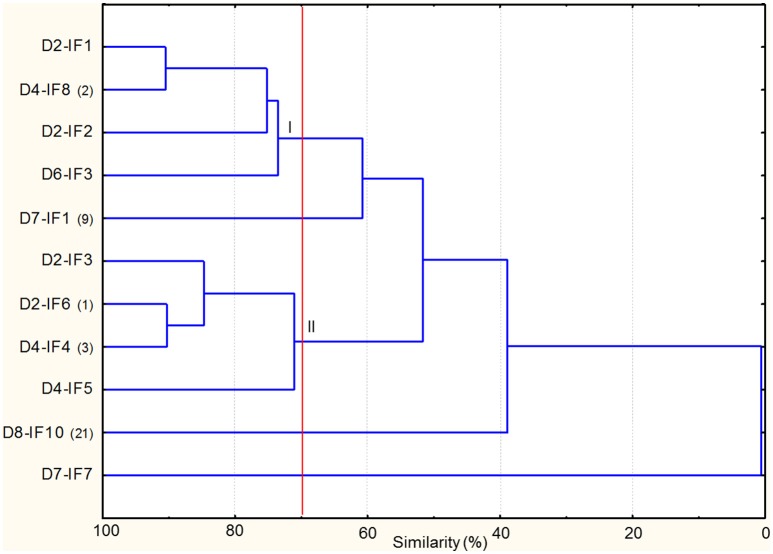
**Dendrogram of combined (primers M13m and RP11) RAPD profiles of ***Saccharomyces cerevisiae*** strains isolated from the mature sourdoughs prepared with irradiated durum wheat flour (IF)**. The alphanumeric code (D2-IF, D4-IF, D6-IF, D7-IF, D8-IF) of the strain name indicates the sourdough of origin. Where present, the numbers in brackets at right of the strain name indicates the number of isolates showing the same RAPD profile. Cluster analysis was based on UPGMA algorithm. Clusters are indicated by Roman numerals (I–II).

As regards yeasts, the combined RAPD profiles were subjected to cluster analysis, which revealed that similarity among strains ranged from ca. 41 to 90% (Figure 3). At 70% of similarity, nine strains, all identified as *S. cerevisiae*, were grouped in two clusters (I–II), with two strains unclustered. Overall, the clustering of the strains was not related to the sourdough of origin. D8-IF10 shared the same RAPD profiles with three, five, and four isolates from D4-IF, D6-IF, and D7-IF sourdoughs (Supplementary Table 6). In addition this strain had the same RAPD profiles as the inoculated strain (*S. cerevisiae* SDA1) (data not shown).

### Bacteria associated to doughs and sourdoughs

RNA extracted from the non-inoculated doughs C-IF and C and from the sourdoughs after five back-slopping steps was used as template for 16S metagenetics analysis in order to describe the bacterial diversity. A total of 377,100 quality-trimmed sequences of *16S rRNA* gene amplicons were obtained. Except for the dough C-IF after the first fermentation, which was characterized by the lowest number of reads (ca. 7000), the number of sequences per sample was higher than 25,000. The average length of the sequences was 383 bp. The highest (*P* <0.05) number of OTU and values of α-diversity indexes were found for the doughs after the first fermentation (C-IF-1st and C-1st) (Supplementary Table 7). Among the sourdoughs, the control (C) showed the highest number of OTU and α-diversity indexes.

**Figure 4:**
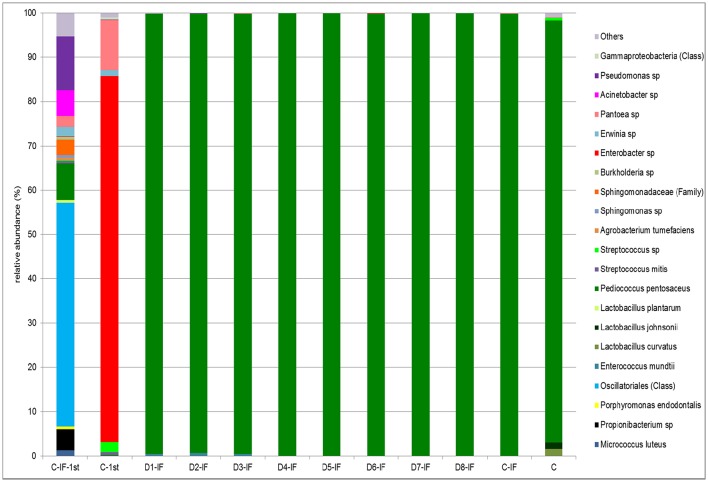
**Relative abundance (%) of bacterial OTUs classified at the highest possible taxonomic level (species/genus/family/class) found in the mature sourdoughs prepared with irradiated (IF) or non-irradiated durum wheat flour (C), and in non-inoculated doughs, after the first fermentation, prepared with irradiated (C-IF-1st) or non-irradiated (C-1st) flour**. Only OTUs with a relative abundance ≥ 0.5% are shown.

Doughs C-IF-1st and C-1st analyzed after the first fermentation were dominated by *Cyanobacteri*a and *Proteobacteria*, respectively. *Firmicutes* were detected in both the doughs at low relative abundance (3.5–10.5%). As expected, upon continuous back-slopping, *Firmicutes* became dominant (> 99.6%) in the C-IF and C sourdoughs, as well as in all the others (data not shown). The relative abundance at the highest possible taxonomic level (species/genus/family/class) is shown in Figure 4. In the C-IF-1st dough, *Oscillatoriales* (class) was the OTU found at the highest relative abundance (50.4%), followed by *Pseudomonas* sp. (12.1%), and *P. pentosaceus* (8.2%). On the contrary, the bacterial community of the dough prepared with non-irradiated flour was dominated, after the first fermentation, by *Enterobacter* sp. (82.6%). *Pantoea* sp. was detected in this dough as subdominant OTU (11.3%). The bacterial community of mature sourdoughs was dominated (> 95.2%) by *P. pentosaceus*, regardless of the use of IF and microbial inoculation. Only the C sourdough harbored *L. curvatus* and *Lactobacillus johnsonii* as minor OTUs with relative abundance of 1.6 and 1.4%, respectively.

### Correlations between microbiota and biochemical characteristics of sourdoughs

**Figure 5:**
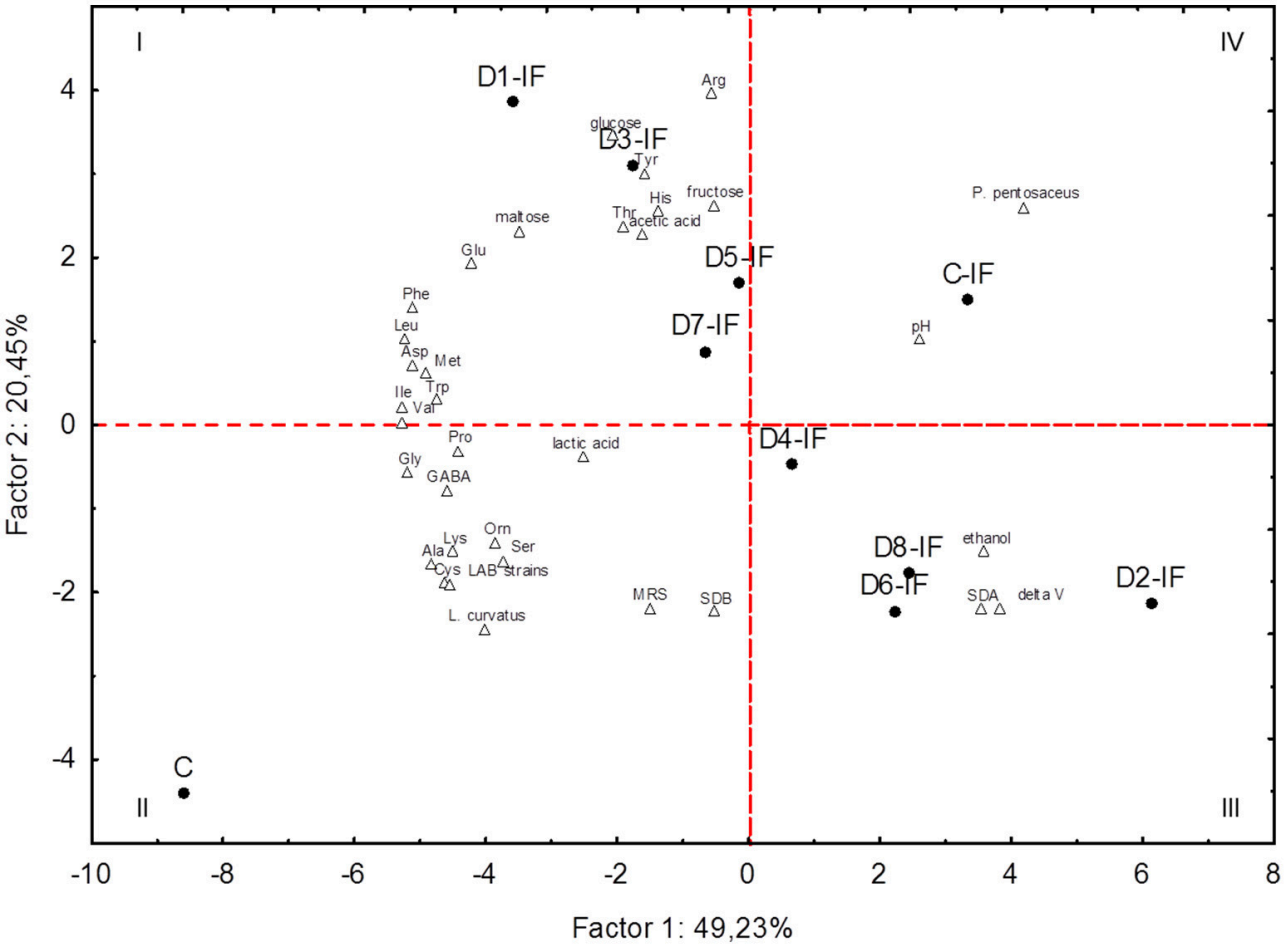
**Score and loading plots of first and second principal components after Principal Component Analysis on pH, leavening capacity (delta V), number of lactic acid bacterium strains (LAB strains), percentage of isolates allotted to ***Lactobacillus curvatus*** (***L. curvatus***) or ***Pediococcus pentosaceus*** (***P. pentosaceus***), cell density of lactic acid bacteria enumerated on MRS (MRS) or SDB (SDB), cell density of yeasts (SDA), concentrations of lactic acid, acetic acid, ethanol, individual free amino acids, maltose, glucose, and fructose in the sourdoughs prepared with irradiated (IF) or non-irradiated durum wheat flour (C)**.

PCA based on the microbiota (cell densities of LAB and yeasts, number of strains, percentage of isolates identified as *L. curvatus* or *P. pentosaceus*) and biochemical characteristics (leavening capacity, pH, lactic and acetic acids, ethanol, carbohydrates, and individual FAA) of sourdoughs clearly differentiated the two control sourdoughs (C and C-IF) from each other and from the inoculated sourdoughs (Figure 5). Two principal components (PC1 and PC2) explained almost 70% of the total variance of the data. The C sourdough fell in the II quadrant because it was characterized mainly by the presence of *L. curvatus* and the highest concentration of several individual FAA. All the sourdoughs (D2-IF, D4-IF, D6-IF, and D8-IF) sharing *S. cerevisiae* SDA1 among the inoculated microorganisms were grouped in the III quadrant, because of their highest leavening capacity and yeast cell density. D1-IF, D3-IF, D5-IF, and D7-IF sourdoughs, characterized by highest concentration of glucose, maltose, arg, and tyr, were grouped in the I quadrant. Among the highest positive variable correlations, concentrations of ala and lys were correlated with *L. curvatus* (*r* = 0.96 and 0.91, respectively). Residual maltose was negatively correlated with ΔV (*r* = −0.95) and ethanol (*r* = −0.96).

## Discussion

Overall, irradiation of flour caused total inactivation of yeasts and a decrease of all the other microbial populations. A reduction (3–6 log cycles) in the total aerobic counts upon similar treatment was reported by other authors (Hanis et al., 1988; Aziz et al., 2006). However, the acidification occurring in the dough C-IF, obtained using IF at all steps and without any inoculated microorganism, could be due to metabolic activity of *P. pentosaceus* that had survived gamma-irradiation. Overall, LAB are more tolerant than *Enterobacteriaceae* to mild (2.5–3 kGy) irradiation treatments (Velasco et al., 2011). *Pediococcus halophilus* survived at ca. 1 log cfu g^−1^ after irradiation of dried jewfish at 3 kGy, but not at 5 kGy (Ito and Yusop Abu, 1985). *Enterococcus* sp. and *Clostridium* sp. survived after 10 kGy treatment of cereal grains (Hanis et al., 1988; Aziz et al., 2006). No differences were found among mature sourdoughs in terms of cell density of LAB, whereas the sourdoughs initially inoculated with the autochthonous strain *S. cerevisiae* SDA1 (D2-IF, D4-IF, D6-IF, D8-IF) had higher yeast cell density than all the others, which allowed dough leavening. These sourdoughs also showed the lowest concentrations of residual glucose and maltose. This could be due to the preference by *S. cerevisiae* toward glucose and maltose (Collar, 1996), coupled to the carbon catabolite repression exerted by glucose (Gänzle and Gobbetti, 2013).

*P. pentosaceus* was the dominant LAB species in all the IF-sourdoughs. This coccus-shaped, facultatively heterofermentative species is characteristic, along with other coccus-shaped LAB, of the first phase of sourdough preparation (Corsetti et al., 2007). Although often referred to as subdominant (Corsetti et al., 2007), *P. pentosaceus* was found at high numbers in some sourdoughs (Kitahara et al., 2005; Aslam et al., 2006; Catzeddu et al., 2006; Scheirlinck et al., 2007; Robert et al., 2009; Minervini et al., 2010, 2012b). Durum wheat flour may be contaminated by *P. pentosaceus* (Alfonzo et al., 2013). Unexpectedly, the autochthonous strain of *P. pentosaceus*, used in all the inoculated doughs, was not retrieved in any sourdough. This could be explained by the low level of inoculum. In addition, this strain may have been outcompeted by other strains of the same species that had recovered from the irradiation damage (Minervini et al., 2010). IF-sourdoughs broadly differed from each other in terms of strains of *P. pentosaceus*. It may be hypothesized that such differences could be attributed to the different microorganisms initially inoculated.

Compared to the IF-sourdoughs, the C sourdough, prepared using non-irradiated flour, differed in terms of number, type and species allotment of strains. Indeed, in this sourdough *L. curvatus* seemed to dominate over *P. pentosaceus*. *L. curvatus* may be encountered in wheat sourdoughs (Zotta et al., 2008; Lattanzi et al., 2013). This result would suggest that irradiation of flour lowered and modified biodiversity of sourdough ecosystem.

Sourdoughs were characterized by different profiles of FAA. The highest concentration of FAA was found for the C sourdough, characterized by the highest number of bacterial strains. It is probable that peptidase activities of different strains were complementary, driving to high degree of proteolysis (De Angelis et al., 2007). As shown by PCA, a strong correlation was found between the presence of *L. curvatus* and concentration of most individual FAA, especially ala and lys. The contribution of this species to proteolysis was reported by other authors in sausage (Fadda et al., 2010; Paredi et al., 2013; López et al., 2015) and sourdough (Zotta et al., 2008). High concentrations of ala and lys were found in food matrices started with *L. curvatus* (Fadda et al., 1999; Ordóñez et al., 1999; Candogan et al., 2009; Chen et al., 2015). Within inoculated IF-sourdoughs, the different (quantitatively and qualitatively) concentrations of FAA could be due to the differences at the level of microbial community. For instance, the lower concentration of FAA found in the D2-IF, D4-IF, D6-IF, and D8-IF sourdoughs, with respect to the others, could be due to consumption of FAA by yeasts (Gobbetti et al., 1994). In addition, different bacterial strains may have caused qualitative differences of FAA, such as in the case of D5-IF and D7-IF sourdoughs that contained diverse concentrations of thr and his.

Overall, the results of 16S metagenetic analysis were in agreement with those from the culture-dependent analysis, except for the C sourdough, whose bacterial microbiota seemed to be dominated by *P. pentosaceus*, rather than *L. curvatus*. In addition, 16S metagenetics performed on the non-inoculated IF-based dough after the first fermentation (C-IF-1st) showed that irradiation had massively inactivated contaminating bacteria, as indicated by the relatively low number of reads. Irradiation also modified bacterial diversity. Indeed, C-IF-1st and non-inoculated, non-irradiated flour-based dough after the first fermentation (C-1st) shared only two (*Erwinia* sp. and *Pantoea* sp.) out of 16 (C-IF-1st) and 7 (C-1st) most representative OTUs.

In conclusion, this work showed that the initial inoculation of dough ecosystem with different combinations of flour autochthonous microorganisms affected the sourdough microbial community in terms of strains of LAB. This, in turn, influenced the profile and concentration of FAA in sourdough. In addition, irradiation of flour at 10 kGy lowered and modified microbial diversity of sourdough ecosystem, but flour autochthonous *P. pentosaceus* strains were able to recover from irradiation damage and to dominate sourdough. Future studies on microbial assembly of sourdough should take into account: (i) the need for minimizing the influence of bacteria resistant to irradiation; (ii) additional combinations of microorganisms (for instance also combinations excluding LAB); and (iii) repeated low level inoculation at each fermentation step.

## Author contributions

GC carried out the experiments, MD discussed the results and wrote the manuscript, FM directed the experimental phases and wrote the manuscript, MG ideated the study and made funds available for the research costs.

### Conflict of interest statement

The authors declare that the research was conducted in the absence of any commercial or financial relationships that could be construed as a potential conflict of interest.
