# Supplementary material for: Different Flour Microbial Communities Drive to Sourdoughs Characterized by Diverse Bacterial Strains and Free Amino Acid Profiles
Source: Front Microbiol. 2016 Nov 8;7:1770. doi: 10.3389/fmicb.2016.01770 (PMC5099235; doi:10.3389/fmicb.2016.01770)
Supplement: Supplementary file 1 [file Table1.DOC]

Supplementary Material

**Different flour microbial communities drive to sourdoughs characterized by diverse bacterial strains and free amino acid profiles**

**Giuseppe Celano, Maria De Angelis, Fabio Minervini*, Marco Gobbetti**

*** Correspondence:** Corresponding Author: fabio.minervini@uniba.it

**Table S1.** Mean values of ΔpH and volume increase (ΔV, in ml) found during the first fermentation (1^st^, carried out at 30 °C for 8 h) and the five consecutive back-slopping steps (I, II, III, IV, V, carried out at 30 °C for 6 h) of doughs prepared with irradiated durum wheat flour (IF) or non-irradiated durum wheat flour (C).

| Dough | 1^st^ | | I | | II | | III | | IV | | V | |
| --- | --- | --- | --- | --- | --- | --- | --- | --- | --- | --- | --- | --- |
|  | ΔpH | ΔV | ΔpH | ΔV | ΔpH | ΔV | ΔpH | ΔV | ΔpH | ΔV | ΔpH | ΔV |
| D1-IF | 0 | 0 | 1.45 (0.06)^b^ | 0 | 1.18 (0.05)^hi^ | 0 | 1.03 (0.02)^mn^ | 0 | 1.00 (0.06)^no^ | 0 | 1.00 (0.01)^no^ | 0 |
| D2-IF | 0 | 0 | 1.46 (0.05)^b^ | 0 | 1.15 (0.03)^i^ | 0 | 0.99 (0.02)^o^ | 2^d^ | 0.95 (0.03)^pq^ | 15 (2)^b^ | 0.93 (0.03)^qr^ | 20 (3)^a^ |
| D3-IF | 0 | 0 | 1.48 (0.06)^b^ | 0 | 1.14 (0.05)^ij^ | 0 | 1.01 (0.00)^no^ | 0 | 0.99 (0.01)^o^ | 0 | 0.99 (0.03)^o^ | 0 |
| D4-IF | 0.02 (0.01)^wx^ | 0 | 1.44 (0.03)^b^ | 0 | 1.16 (0.01)^i^ | 0 | 0.98 (0.03)^op^ | 0 | 0.99 (0.05)^o^ | 0 | 0.99 (0.02)^o^ | 10 (1)^c^ |
| D5-IF | 0.01 (0.00)^x^ | 0 | 0.89 (0.03)^r^ | 0 | 1.26 (0.06)^fg^ | 0 | 1.27 (0.02)^ef^ | 0 | 0.98 (0.02)^op^ | 0 | 0.93 (0.01)^qr^ | 0 |
| D6-IF | 0.05 (0.01)^vw^ | 0 | 1.32 (0.04)^cd^ | 4 (0)^d^ | 1.59 (0.04)^a^ | 0 | 1.22 (0.01)^gh^ | 0 | 1.04 (0.01)^mn^ | 5 (1)^d^ | 1.03 (0.04)^mn^ | 12 (1)^bc^ |
| D7-IF | 0 | 0 | 1.28 (0.03)^de^ | 2 (0)^d^ | 1.36 (0.02)^c^ | 0 | 1.00 (0.05)^no^ | 0 | 1.12 (0.02)^ij^ | 0 | 1.08 (0.03)^kl^ | 0 |
| D8-IF | 0.14 (0.00)^u^ | 0 | 1.14 (0.02)^ij^ | 5 (1)^d^ | 1.46 (0.06)^b^ | 0 | 1.11 (0.03)^jk^ | 0 | 1.17 (0.04)^i^ | 3 (1)^d^ | 1.09 (0.05)^kl^ | 10 (2)^c^ |
| C-IF*^a^* | 0 | 0 | 0.09 (0.02)^v^ | 0 | 0 | 0 | 1.00 (0.02)^no^ | 0 | 0.80 (0.01)^s^ | 0 | 0.90 (0.01)^r^ | 0 |
| C*^b^* | 0 | 5^d^ | 0.10 (0.02)^uv^ | 5 (0)^d^ | 1.60 (0.01)^a^ | 0 | 1.10 (0.00)^jk^ | 0 | 0.76 (0.02)^s^ | 0 | 0.70 (0.03)^s^ | 0 |

*^a^* Dough prepared with irradiated flour, without inoculation; *^b^* Dough prepared with non-irradiated flour, without inoculation.
