# Supplementary material for: Different Flour Microbial Communities Drive to Sourdoughs Characterized by Diverse Bacterial Strains and Free Amino Acid Profiles
Source: Front Microbiol. 2016 Nov 8;7:1770. doi: 10.3389/fmicb.2016.01770 (PMC5099235; doi:10.3389/fmicb.2016.01770)
Supplement: Supplementary file 2 [file Table2.DOCX]

***Supplementary Material***

**Different flour microbial communities drive to sourdoughs characterized by diverse bacterial strains and free amino acid profiles**

**Giuseppe Celano, Maria De Angelis, Fabio Minervini*, Marco Gobbetti**

*** Correspondence:** Corresponding Author: fabio.minervini@uniba.it

**Table S2.** Mean values (standard deviation) of cell density (log cfu g^-1^) of presumptive lactic acid bacteria (enumerated on MRS), yeasts and *Enterobacteriaceae* found after the first fermentation (1^st^, carried out at 30 °C for 8 h) and the four consecutive back-slopping steps (I, II, III, IV, carried out at 30 °C for 6 h) of doughs prepared with irradiated durum wheat flour (IF) or non-irradiated durum wheat flour (C).

| Dough | Lactic acid bacteria | | | | | Yeasts | | | | | *Enterobacteriaceae* | | | | |
| --- | --- | --- | --- | --- | --- | --- | --- | --- | --- | --- | --- | --- | --- | --- | --- |
|  | 1^st^ | I | II | III | IV | 1^st^ | I | II | III | IV | 1^st^ | I | II | III | IV |
| D1-IF | 3.6 (0.1) | 4.9 (0.2) | 6.0 (0.1) | 6.8 (0.1) | 7.9 (0.0) | 0.6 (0.4) | 1.0 (0.2) | 0.8 (0.2) | 0.5 (0.5) | 0.4 (0.6) | 1.0 (0.3) | 0.0 | 0.0 | 0.0 | 0.0 |
| D2-IF | 3.6 (0.2) | 5.0 (0.0) | 5.9 (0.2) | 7.0 (0.1) | 8.1 (0.1) | 2.4 (0.2) | 3.3 (0.3) | 4.1 (0.1) | 5.0 (0.1) | 5.9 (0.2) | 1.3 (0.1) | 0.0 | 0.0 | 0.0 | 0.0 |
| D3-IF | 3.3 (0.1) | 4.8 (0.2) | 6.3 (0.2) | 7.0 (0.1) | 7.8 (0.1) | 0.5 (0.4) | 0.8 (0.2) | 0.5 (0.5) | 0.6 (0.4) | 0.3 (0.6) | 2.8 (0.2) | 0.7 (0.3) | 0.0 | 0.0 | 0.0 |
| D4-IF | 3.4 (0.1) | 4.9 (0.1) | 6.0 (0.1) | 6.9 (0.2) | 7.8 (0.3) | 2.4 (0.2) | 3.2 (0.3) | 4.1 (0.1) | 4.8 (0.1) | 5.6 (0.4) | 3.0 (0.4) | 0.0 | 0.0 | 0.0 | 0.0 |
| D5-IF | 3.3 (0.0) | 4.4 (0.1) | 6.1 (0.1) | 7.0 (0.1) | 7.7 (0.1) | 0.8 (0.3) | 1.0 (0.1) | 0.6 (0.4) | 0.3 (0.6) | 0.5 (0.5) | 2.6 (0.0) | 2.1 (0.2) | 0.7 (0.3) | 0.0 | 0.0 |
| D6-IF | 3.4 (0.1) | 4.5 (0.3) | 5.9 (0.2) | 6.8 (0.0) | 8.0 (0.1) | 2.5 (0.0) | 3.0 (0.1) | 3.8 (0.3) | 4.7 (0.0) | 5.6 (0.2) | 3.0 (0.1) | 2.5 (0.4) | 0.3 (0.5) | 0.0 | 0.0 |
| D7-IF | 3.6 (0.1) | 4.4 (0.2) | 6.3 (0.0) | 7.1 (0.1) | 7.9 (0.2) | 0.5 (0.5) | 0.9 (0.2) | 1.3 (0.2) | 1.9 (0.4) | 2.5 (0.2) | 2.9 (0.1) | 2.2 (0.1) | 0.0 | 0.0 | 0.0 |
| D8-IF | 3.5 (0.2) | 4.5 (0.1) | 5.8 (0.0) | 6.9 (0.3) | 8.0 (0.1) | 2.2 (0.3) | 2.9 (0.1) | 3.6 (0.0) | 4.5 (0.4) | 5.3 (0.2) | 2.7 (0.4) | 2.0 (0.2) | 0.0 | 0.0 | 0.0 |
| C-IF | 2.0 (0.2) | 3.1 (0.2) | 4.0 (0.1) | 5.3 (0.1) | 6.7 (0.2) | 0.9 (0.1) | 0.6 (0.4) | 0.8 (0.3) | 0.3 (0.6) | 0.3 (0.5) | 1.1 (0.1) | 1.4 (0.1) | 1.9 (0.3) | 1.0 (0.2) | 0.7 (0.3) |
| C | 2.8 (0.1) | 4.2 (0.0) | 5.5 (0.1) | 6.4 (0.2) | 7.2 (0.2) | 1.6 (0.1) | 1.7 (0.1) | 1.2 (0.3) | 0.9 (0.2) | 0.4 (0.6) | 3.5 (0.2) | 2.5 (0.4) | 0.8 (0.2) | 0.0 | 0.0 |
