# Supplementary material for: Different Flour Microbial Communities Drive to Sourdoughs Characterized by Diverse Bacterial Strains and Free Amino Acid Profiles
Source: Front Microbiol. 2016 Nov 8;7:1770. doi: 10.3389/fmicb.2016.01770 (PMC5099235; doi:10.3389/fmicb.2016.01770)
Supplement: Supplementary file 3 [file Table3.DOC]

Supplementary Material

**Different flour microbial communities drive to sourdoughs characterized by diverse bacterial strains and free amino acid profiles**

**Giuseppe Celano, Maria De Angelis, Fabio Minervini*, Marco Gobbetti**

*** Correspondence:** Corresponding Author: [fabio.minervini@uniba.it](mailto:fabio.minervini@uniba.it)

**TABLE S3.** Concentrations of individual free amino acids (FAA) [mg kg^−1^] in the mature sourdoughs prepared with irradiated durum wheat flour (IF) or non-irradiated flour (C).

| Sourdoughs | Asp | Thr | Ser | Glu | Gly | Ala | Cys | Val | Met | Ile | Leu | Tyr | Phe | GABA | His | Trp | Orn | Lys | Arg | Pro |
| --- | --- | --- | --- | --- | --- | --- | --- | --- | --- | --- | --- | --- | --- | --- | --- | --- | --- | --- | --- | --- |
| D1-IF | 123 | 22 | 21 | 158 | 36 | 45 | 40 | 57 | 31 | 52 | 117 | 41 | 70 | 61 | 45 | 88 | 14 | 28 | 62 | 59 |
| D2-IF | 15 | 5 | 18 | 66 | 14 | 18 | 24 | 6 | 2 | 5 | 13 | 2 | 4 | 3 | 33 | 12 | 2 | 25 | 38 | 32 |
| D3-IF | 114 | 76 | 13 | 140 | 30 | 37 | 35 | 44 | 13 | 34 | 100 | 36 | 62 | 48 | 40 | 81 | 17 | 36 | 59 | 57 |
| D4-IF | 37 | 4 | 12 | 74 | 29 | 44 | 40 | 43 | 6 | 29 | 56 | 30 | 38 | 58 | 31 | 81 | 18 | 17 | 32 | 60 |
| D5-IF | 103 | 71 | 12 | 107 | 25 | 30 | 29 | 34 | 9 | 25 | 73 | 27 | 49 | 66 | 41 | 78 | 13 | 27 | 51 | 51 |
| D6-IF | 25 | 3 | 7 | 50 | 24 | 34 | 35 | 32 | 6 | 23 | 40 | 25 | 28 | 53 | 28 | 75 | 18 | 18 | 25 | 62 |
| D7-IF | 99 | 58 | 4 | 109 | 26 | 31 | 34 | 39 | 14 | 29 | 80 | 30 | 50 | 57 | 22 | 78 | 11 | 26 | 50 | 63 |
| D8-IF | 22 | 5 | 10 | 47 | 25 | 32 | 39 | 32 | 7 | 22 | 38 | 25 | 20 | 49 | 30 | 71 | 19 | 21 | 19 | 49 |
| C-IF*^a^* | 44 | 39 | 5 | 40 | 13 | 23 | 25 | 18 | 5 | 12 | 29 | 13 | 19 | 64 | 34 | 58 | 1 | 13 | 44 | 42 |
| C*^b^* | 185 | 35 | 39 | 130 | 48 | 95 | 66 | 72 | 30 | 60 | 131 | 10 | 73 | 123 | 33 | 112 | 26 | 72 | 23 | 71 |

*^a^* Dough prepared with irradiated flour, without inoculation

*^b^* Dough prepared with non-irradiated flour, without inoculation
