# Supplementary material for: Different Flour Microbial Communities Drive to Sourdoughs Characterized by Diverse Bacterial Strains and Free Amino Acid Profiles
Source: Front Microbiol. 2016 Nov 8;7:1770. doi: 10.3389/fmicb.2016.01770 (PMC5099235; doi:10.3389/fmicb.2016.01770)
Supplement: Supplementary file 4 [file Table4.DOC]

Supplementary Material

**Different flour microbial communities drive to sourdoughs characterized by diverse bacterial strains and free amino acid profiles**

**Giuseppe Celano, Maria De Angelis, Fabio Minervini*, Marco Gobbetti**

*** Correspondence:** Corresponding Author: [fabio.minervini@uniba.it](mailto:fabio.minervini@uniba.it)

**TABLE S4.** Presumptive lactic acid bacteria (LAB) and yeasts (Y) isolated from the mature sourdoughs prepared with irradiated durum wheat flour (IF) or non-irradiated flour (C).

| **Isolate** | **Sourdough^a^** | **Medium of isolation** | **Dilution of isolation** |
| --- | --- | --- | --- |
| (LAB) C A3 | C | MRS | 10^-7^ |
| (LAB) C A4  (LAB) C A5 | C | MRS | 10^-7^ |
|  | C | MRS | 10^-7^ |
| (LAB) C A6 | C | MRS | 10^-7^ |
| (LAB) C A7 | C | MRS | 10^-7^ |
| (LAB) C A8 | C | MRS | 10^-7^ |
| (LAB) C A9 | C | MRS | 10^-7^ |
| (LAB) C A10 | C | MRS | 10^-7^ |
| (LAB) C A11 | C | MRS | 10^-7^ |
| (LAB) C A12 | C | MRS | 10^-7^ |
| (LAB) C A13 | C | MRS | 10^-7^ |
| (LAB) C A14 | C | MRS | 10^-7^ |
| (LAB) B 1 | C | SDB | 10^-7^ |
| (LAB) B 2 | C | SDB | 10^-7^ |
| (LAB) B 4 | C | SDB | 10^-7^ |
| (LAB) B 5 | C | SDB | 10^-7^ |
| (LAB) B 6 | C | SDB | 10^-7^ |
| (LAB) B 7 | C | SDB | 10^-7^ |
| (LAB) B 8 | C | SDB | 10^-7^ |
| (LAB) B 9 | C | SDB | 10^-7^ |
| (LAB) B 11 | C | SDB | 10^-7^ |
| (LAB) B 12 | C | SDB | 10^-7^ |
| (LAB) B 13 | C | SDB | 10^-7^ |
| (LAB) B 14 | C | SDB | 10^-7^ |
| (LAB) B 15 | C | SDB | 10^-7^ |
| (LAB) C-IF A1 | C-IF | MRS | 10^-7^ |
| (LAB) C-IF A2 | C-IF | MRS | 10^-7^ |
| (LAB) C-IF A3 | C-IF | MRS | 10^-7^ |
| (LAB) C-IF A4 | C-IF | MRS | 10^-7^ |
| (LAB) C-IF A5 | C-IF | MRS | 10^-7^ |
| (LAB) C-IF A6 | C-IF | MRS | 10^-7^ |
| (LAB) C-IF A7 | C-IF | MRS | 10^-7^ |
| (LAB) C-IF A8 | C-IF | MRS | 10^-7^ |
| (LAB) C-IF A9 | C-IF | MRS | 10^-7^ |
| (LAB) C-IF A10 | C-IF | MRS | 10^-7^ |
| (LAB) C-IF A11 | C-IF | MRS | 10^-7^ |
| (LAB) C-IF A12 | C-IF | MRS | 10^-7^ |
| (LAB) C-IF B2 | C-IF | SDB | 10^-7^ |
| (LAB) C-IF B3 | C-IF | SDB | 10^-7^ |
| (LAB) C-IF B4 | C-IF | SDB | 10^-7^ |
| (LAB) C-IF B5 | C-IF | SDB | 10^-7^ |
| (LAB) C-IF B6 | C-IF | SDB | 10^-7^ |
| (LAB) C-IF B7 | C-IF | SDB | 10^-7^ |
| (LAB) C-IF B8 | C-IF | SDB | 10^-7^ |
| (LAB) C-IF B9 | C-IF | SDB | 10^-7^ |
| (LAB) C-IF B10 | C-IF | SDB | 10^-7^ |
| (LAB) C-IF B11 | C-IF | SDB | 10^-7^ |
| (LAB) C-IF B12 | C-IF | SDB | 10^-7^ |
| (LAB) D1-IF A1 | D1-IF | MRS | 10^-7^ |
| (LAB) D1-IF A2 | D1-IF | MRS | 10^-7^ |
| (LAB) D1-IF A3 | D1-IF | MRS | 10^-7^ |
| (LAB) D1-IF A4 | D1-IF | MRS | 10^-7^ |
| (LAB) D1-IF A5 | D1-IF | MRS | 10^-7^ |
| (LAB) D1-IF A6 | D1-IF | MRS | 10^-7^ |
| (LAB) D1-IF A7 | D1-IF | MRS | 10^-7^ |
| (LAB) D1-IF A8 | D1-IF | MRS | 10^-7^ |
| (LAB) D1-IF A9 | D1-IF | MRS | 10^-7^ |
| (LAB) D1-IF A10 | D1-IF | MRS | 10^-7^ |
| (LAB) D1-IF A11 | D1-IF | MRS | 10^-7^ |
| (LAB) D1-IF A12 | D1-IF | MRS | 10^-7^ |
| (LAB) D1-IF A13 | D1-IF | MRS | 10^-7^ |
| (LAB) D1-IF A14 | D1-IF | MRS | 10^-7^ |
| (LAB) D1-IF A15 | D1-IF | MRS | 10^-7^ |
| (LAB) D1-IF B1 | D1-IF | SDB | 10^-7^ |
| (LAB) D1-IF B2 | D1-IF | SDB | 10^-7^ |
| (LAB) D1-IF B3 | D1-IF | SDB | 10^-7^ |
| (LAB) D1-IF B4 | D1-IF | SDB | 10^-7^ |
| (LAB) D1-IF B5 | D1-IF | SDB | 10^-7^ |
| (LAB) D1-IF B6 | D1-IF | SDB | 10^-7^ |
| (LAB) D1-IF B7 | D1-IF | SDB | 10^-7^ |
| (LAB) D1-IF B8 | D1-IF | SDB | 10^-7^ |
| (LAB) D1-IF B9 | D1-IF | SDB | 10^-7^ |
| (LAB) D1-IF B10 | D1-IF | SDB | 10^-7^ |
| (LAB) D1-IF B11 | D1-IF | SDB | 10^-7^ |
| (LAB) D1-IF B12 | D1-IF | SDB | 10^-7^ |
| (LAB) D1-IF B13 | D1-IF | SDB | 10^-7^ |
| (LAB) D1-IF B14 | D1-IF | SDB | 10^-7^ |
| (LAB) D1-IF B15 | D1-IF | SDB | 10^-7^ |
| (LAB) D2-IF A1 | D2-IF | MRS | 10^-7^ |
| (LAB) D2-IF A2 | D2-IF | MRS | 10^-7^ |
| (LAB) D2-IF A3 | D2-IF | MRS | 10^-7^ |
| (LAB) D2-IF A4 | D2-IF | MRS | 10^-7^ |
| (LAB) D2-IF A5 | D2-IF | MRS | 10^-7^ |
| (LAB) D2-IF A6 | D2-IF | MRS | 10^-7^ |
| (LAB) D2-IF A7 | D2-IF | MRS | 10^-7^ |
| (LAB) D2-IF A8 | D2-IF | MRS | 10^-7^ |
| (LAB) D2-IF A9 | D2-IF | MRS | 10^-7^ |
| (LAB) D2-IF A10 | D2-IF | MRS | 10^-7^ |
| (LAB) D2-IF A13 | D2-IF | MRS | 10^-7^ |
| (LAB) D2-IF B1 | D2-IF | SDB | 10^-7^ |
| (LAB) D2-IF B2 | D2-IF | SDB | 10^-7^ |
| (LAB) D2-IF B3 | D2-IF | SDB | 10^-7^ |
| (LAB) D2-IF B4 | D2-IF | SDB | 10^-7^ |
| (LAB) D2-IF B5 | D2-IF | SDB | 10^-7^ |
| (LAB) D2-IF B6 | D2-IF | SDB | 10^-7^ |
| (LAB) D2-IF B7 | D2-IF | SDB | 10^-7^ |
| (LAB) D2-IF B8 | D2-IF | SDB | 10^-7^ |
| (LAB) D2-IF B9 | D2-IF | SDB | 10^-7^ |
| (LAB) D2-IF B10 | D2-IF | SDB | 10^-7^ |
| (LAB) D2-IF B11 | D2-IF | SDB | 10^-7^ |
| (LAB) D2-IF B12 | D2-IF | SDB | 10^-7^ |
| (LAB) D2-IF B13 | D2-IF | SDB | 10^-7^ |
| (LAB) D2-IF B14 | D2-IF | SDB | 10^-7^ |
| (LAB) D2-IF B15 | D2-IF | SDB | 10^-7^ |
| (Y) D2-IF 1 | D2-IF | SDA^b^ | 10^-5^ |
| (Y) D2-IF 2 | D2-IF | SDA | 10^-5^ |
| (Y) D2-IF 3 | D2-IF | SDA | 10^-5^ |
| (Y) D2-IF 4 | D2-IF | SDA | 10^-5^ |
| (Y) D2-IF 5 | D2-IF | SDA | 10^-5^ |
| (Y) D2-IF 6 | D2-IF | SDA | 10^-5^ |
| (Y) D2-IF 7 | D2-IF | SDA | 10^-5^ |
| (Y) D2-IF 8 | D2-IF | SDA | 10^-5^ |
| (Y) D2-IF 9 | D2-IF | SDA | 10^-5^ |
| (Y) D2-IF 10 | D2-IF | SDA | 10^-5^ |
| (LAB) D3-IF A2 | D3-IF | MRS | 10^-7^ |
| (LAB) D3-IF B1 | D3-IF | SDB | 10^-7^ |
| (LAB) D3-IF B2 | D3-IF | SDB | 10^-7^ |
| (LAB) D3-IF B3 | D3-IF | SDB | 10^-7^ |
| (LAB) D3-IF B5 | D3-IF | SDB | 10^-7^ |
| (LAB) D3-IF B6 | D3-IF | SDB | 10^-7^ |
| (LAB) D3-IF B7 | D3-IF | SDB | 10^-7^ |
| (LAB) D3-IF B8 | D3-IF | SDB | 10^-7^ |
| (LAB) D3-IF B9 | D3-IF | SDB | 10^-7^ |
| (LAB) D3-IF B10 | D3-IF | SDB | 10^-7^ |
| (LAB) D3-IF B11 | D3-IF | SDB | 10^-7^ |
| (LAB) D3-IF B12 | D3-IF | SDB | 10^-7^ |
| (LAB) D3-IF B13 | D3-IF | SDB | 10^-7^ |
| (LAB) D3-IF B14 | D3-IF | SDB | 10^-7^ |
| (LAB) D3-IF B15 | D3-IF | SDB | 10^-7^ |
| (LAB) D4-IF A1 | D4-IF | MRS | 10^-7^ |
| (LAB) D4-IF A2 | D4-IF | MRS | 10^-7^ |
| (LAB) D4-IF A3 | D4-IF | MRS | 10^-7^ |
| (LAB) D4-IF A4 | D4-IF | MRS | 10^-7^ |
| (LAB) D4-IF A5 | D4-IF | MRS | 10^-7^ |
| (LAB) D4-IF A6 | D4-IF | MRS | 10^-7^ |
| (LAB) D4-IF A7 | D4-IF | MRS | 10^-7^ |
| (LAB) D4-IF A8 | D4-IF | MRS | 10^-7^ |
| (LAB) D4-IF A9 | D4-IF | MRS | 10^-7^ |
| (LAB) D4-IF A10 | D4-IF | MRS | 10^-7^ |
| (LAB) D4-IF A11 | D4-IF | MRS | 10^-7^ |
| (LAB) D4-IF A12 | D4-IF | MRS | 10^-7^ |
| (LAB) D4-IF A13 | D4-IF | MRS | 10^-7^ |
| (LAB) D4-IF A14 | D4-IF | MRS | 10^-7^ |
| (LAB) D4-IF A15 | D4-IF | MRS | 10^-7^ |
| (LAB) D4-IF B1 | D4-IF | SDB | 10^-7^ |
| (LAB) D4-IF B2 | D4-IF | SDB | 10^-7^ |
| (LAB) D4-IF B3 | D4-IF | SDB | 10^-7^ |
| (LAB) D4-IF B4 | D4-IF | SDB | 10^-7^ |
| (LAB) D4-IF B5 | D4-IF | SDB | 10^-7^ |
| (LAB) D4-IF B6 | D4-IF | SDB | 10^-7^ |
| (LAB) D4-IF B7 | D4-IF | SDB | 10^-7^ |
| (LAB) D4-IF B8 | D4-IF | SDB | 10^-7^ |
| (LAB) D4-IF B9 | D4-IF | SDB | 10^-7^ |
| (LAB) D4-IF B11 | D4-IF | SDB | 10^-7^ |
| (LAB) D4-IF B12 | D4-IF | SDB | 10^-7^ |
| (LAB) D4-IF B13 | D4-IF | SDB | 10^-7^ |
| (LAB) D4-IF B14 | D4-IF | SDB | 10^-7^ |
| (LAB) D4-IF B15 | D4-IF | SDB | 10^-7^ |
| (Y) D4-IF 1 | D4-IF | SDA | 10^-5^ |
| (Y) D4-IF 2 | D4-IF | SDA | 10^-5^ |
| (Y) D4-IF 3 | D4-IF | SDA | 10^-5^ |
| (Y) D4-IF 4 | D4-IF | SDA | 10^-5^ |
| (Y) D4-IF 5 | D4-IF | SDA | 10^-5^ |
| (Y) D4-IF 6 | D4-IF | SDA | 10^-5^ |
| (Y) D4-IF 7 | D4-IF | SDA | 10^-5^ |
| (Y) D4-IF 8 | D4-IF | SDA | 10^-5^ |
| (Y) D4-IF 9 | D4-IF | SDA | 10^-5^ |
| (Y) D4-IF 10 | D4-IF | SDA | 10^-5^ |
| (LAB) D5-IF A1 | D5-IF | MRS | 10^-7^ |
| (LAB) D5-IF A2 | D5-IF | MRS | 10^-7^ |
| (LAB) D5-IF A3 | D5-IF | MRS | 10^-7^ |
| (LAB) D5-IF A4 | D5-IF | MRS | 10^-7^ |
| (LAB) D5-IF A5 | D5-IF | MRS | 10^-7^ |
| (LAB) D5-IF A7 | D5-IF | MRS | 10^-7^ |
| (LAB) D5-IF A8 | D5-IF | MRS | 10^-7^ |
| (LAB) D5-IF A9 | D5-IF | MRS | 10^-7^ |
| (LAB) D5-IF A10 | D5-IF | MRS | 10^-7^ |
| (LAB) D5-IF A12 | D5-IF | MRS | 10^-7^ |
| (LAB) D5-IF A13 | D5-IF | MRS | 10^-7^ |
| (LAB) D5-IF A14 | D5-IF | MRS | 10^-7^ |
| (LAB) D5-IF A15 | D5-IF | MRS | 10^-7^ |
| (LAB) D5-IF B1 | D5-IF | SDB | 10^-7^ |
| (LAB) D5-IF B2 | D5-IF | SDB | 10^-7^ |
| (LAB) D5-IF B3 | D5-IF | SDB | 10^-7^ |
| (LAB) D5-IF B4 | D5-IF | SDB | 10^-7^ |
| (LAB) D5-IF B5 | D5-IF | SDB | 10^-7^ |
| (LAB) D5-IF B6 | D5-IF | SDB | 10^-7^ |
| (LAB) D5-IF B7 | D5-IF | SDB | 10^-7^ |
| (LAB) D5-IF B8 | D5-IF | SDB | 10^-7^ |
| (LAB) D5-IF B9 | D5-IF | SDB | 10^-7^ |
| (LAB) D5-IF B10 | D5-IF | SDB | 10^-7^ |
| (LAB) D5-IF B11 | D5-IF | SDB | 10^-7^ |
| (LAB) D5-IF B12 | D5-IF | SDB | 10^-7^ |
| (LAB) D5-IF B13 | D5-IF | SDB | 10^-7^ |
| (LAB) D5-IF B14 | D5-IF | SDB | 10^-7^ |
| (LAB) D5-IF B15 | D5-IF | SDB | 10^-7^ |
| (LAB) D6-IF A1 | D6-IF | MRS | 10^-7^ |
| (LAB) D6-IF A2 | D6-IF | MRS | 10^-7^ |
| (LAB) D6-IF A3 | D6-IF | MRS | 10^-7^ |
| (LAB) D6-IF A4 | D6-IF | MRS | 10^-7^ |
| (LAB) D6-IF A5 | D6-IF | MRS | 10^-7^ |
| (LAB) D6-IF A6 | D6-IF | MRS | 10^-7^ |
| (LAB) D6-IF A7 | D6-IF | MRS | 10^-7^ |
| (LAB) D6-IF A8 | D6-IF | MRS | 10^-7^ |
| (LAB) D6-IF A9 | D6-IF | MRS | 10^-7^ |
| (LAB) D6-IF A10 | D6-IF | MRS | 10^-7^ |
| (LAB) D6-IF A11 | D6-IF | MRS | 10^-7^ |
| (LAB) D6-IF A12 | D6-IF | MRS | 10^-7^ |
| (LAB) D6-IF A13 | D6-IF | MRS | 10^-7^ |
| (LAB) D6-IF A14 | D6-IF | MRS | 10^-7^ |
| (LAB) D6-IF A15 | D6-IF | MRS | 10^-7^ |
| (LAB) D6-IF B1 | D6-IF | SDB | 10^-7^ |
| (LAB) D6-IF B2 | D6-IF | SDB | 10^-7^ |
| (LAB) D6-IF B3 | D6-IF | SDB | 10^-7^ |
| (LAB) D6-IF B4 | D6-IF | SDB | 10^-7^ |
| (LAB) D6-IF B5 | D6-IF | SDB | 10^-7^ |
| (LAB) D6-IF B6 | D6-IF | SDB | 10^-7^ |
| (LAB) D6-IF B7 | D6-IF | SDB | 10^-7^ |
| (LAB) D6-IF B8 | D6-IF | SDB | 10^-7^ |
| (LAB) D6-IF B9 | D6-IF | SDB | 10^-7^ |
| (LAB) D6-IF B10 | D6-IF | SDB | 10^-7^ |
| (LAB) D6-IF B11 | D6-IF | SDB | 10^-7^ |
| (LAB) D6-IF B12 | D6-IF | SDB | 10^-7^ |
| (LAB) D6-IF B13 | D6-IF | SDB | 10^-7^ |
| (LAB) D6-IF B14 | D6-IF | SDB | 10^-7^ |
| (LAB) D6-IF B15 | D6-IF | SDB | 10^-7^ |
| (Y) D6-IF 1 | D6-IF | SDA | 10^-5^ |
| (Y) D6-IF 2 | D6-IF | SDA | 10^-5^ |
| (Y) D6-IF 3 | D6-IF | SDA | 10^-5^ |
| (Y) D6-IF 4 | D6-IF | SDA | 10^-5^ |
| (Y) D6-IF 5 | D6-IF | SDA | 10^-5^ |
| (Y) D6-IF 6 | D6-IF | SDA | 10^-5^ |
| (Y) D6-IF 7 | D6-IF | SDA | 10^-5^ |
| (Y) D6-IF 8 | D6-IF | SDA | 10^-5^ |
| (Y) D6-IF 9 | D6-IF | SDA | 10^-5^ |
| (Y) D6-IF 10 | D6-IF | SDA | 10^-5^ |
| (LAB) D7-IF A1 | D7-IF | MRS | 10^-7^ |
| (LAB) D7-IF A2 | D7-IF | MRS | 10^-7^ |
| (LAB) D7-IF A3 | D7-IF | MRS | 10^-7^ |
| (LAB) D7-IF A9 | D7-IF | MRS | 10^-7^ |
| (LAB) D7-IF A10 | D7-IF | MRS | 10^-7^ |
| (LAB) D7-IF A11 | D7-IF | MRS | 10^-7^ |
| (LAB) D7-IF A12 | D7-IF | MRS | 10^-7^ |
| (LAB) D7-IF A13 | D7-IF | MRS | 10^-7^ |
| (LAB) D7-IF A14 | D7-IF | MRS | 10^-7^ |
| (LAB) D7-IF A15 | D7-IF | MRS | 10^-7^ |
| (Y) D7-IF B1 | D7-IF | SDB | 10^-7^ |
| (Y) D7-IF 1 | D7-IF | SDA | 10^-1^ |
| (Y) D7-IF 2 | D7-IF | SDA | 10^-1^ |
| (Y) D7-IF 4 | D7-IF | SDA | 10^-1^ |
| (Y) D7-IF 5 | D7-IF | SDA | 10^-1^ |
| (Y) D7-IF 7 | D7-IF | SDA | 10^-1^ |
| (Y) D7-IF 8 | D7-IF | SDA | 10^-1^ |
| (Y) D7-IF 10 | D7-IF | SDA | 10^-1^ |
| (LAB) D8-IF A1 | D8-IF | MRS | 10^-7^ |
| (LAB) D8-IF A2 | D8-IF | MRS | 10^-7^ |
| (LAB) D8-IF A3 | D8-IF | MRS | 10^-7^ |
| (LAB) D8-IF A4 | D8-IF | MRS | 10^-7^ |
| (LAB) D8-IF A5 | D8-IF | MRS | 10^-7^ |
| (LAB) D8-IF A6 | D8-IF | MRS | 10^-7^ |
| (LAB) D8-IF A7 | D8-IF | MRS | 10^-7^ |
| (LAB) D8-IF A8 | D8-IF | MRS | 10^-7^ |
| (LAB) D8-IF A9 | D8-IF | MRS | 10^-7^ |
| (LAB) D8-IF A10 | D8-IF | MRS | 10^-7^ |
| (LAB) D8-IF A11 | D8-IF | MRS | 10^-7^ |
| (LAB) D8-IF A12 | D8-IF | MRS | 10^-7^ |
| (LAB) D8-IF A13 | D8-IF | MRS | 10^-7^ |
| (LAB) D8-IF A14 | D8-IF | MRS | 10^-7^ |
| (LAB) D8-IF A15 | D8-IF | MRS | 10^-7^ |
| (Y) D8-IF 1 | D8-IF | SDA | 10^-5^ |
| (Y) D8-IF 2 | D8-IF | SDA | 10^-5^ |
| (Y) D8-IF 3 | D8-IF | SDA | 10^-5^ |
| (Y) D8-IF 4 | D8-IF | SDA | 10^-5^ |
| (Y) D8-IF 5 | D8-IF | SDA | 10^-5^ |
| (Y) D8-IF 6 | D8-IF | SDA | 10^-5^ |
| (Y) D8-IF 7 | D8-IF | SDA | 10^-5^ |
| (Y) D8-IF 8 | D8-IF | SDA | 10^-5^ |
| (Y) D8-IF 9 | D8-IF | SDA | 10^-5^ |
| (Y) D8-IF 10 | D8-IF | SDA | 10^-5^ |

^a^ Sourdoughs are coded according to the Table 2.

^b^ SDA, Sabouraud Dextrose Agar.
