# Supplementary material for: Different Flour Microbial Communities Drive to Sourdoughs Characterized by Diverse Bacterial Strains and Free Amino Acid Profiles
Source: Front Microbiol. 2016 Nov 8;7:1770. doi: 10.3389/fmicb.2016.01770 (PMC5099235; doi:10.3389/fmicb.2016.01770)
Supplement: Supplementary file 5 [file Table5.DOC]

Supplementary Material

**Different flour microbial communities drive to sourdoughs characterized by diverse bacterial strains and free amino acid profiles**

**Giuseppe Celano, Maria De Angelis, Fabio Minervini*, Marco Gobbetti**

*** Correspondence:** Corresponding Author: [fabio.minervini@uniba.it](mailto:fabio.minervini@uniba.it)

**TABLE S5.** Species and strains of lactic acid bacteria isolated from the mature sourdoughs prepared with irradiated (IF) or non-irradiated durum wheat flour (C).

| **Sourdough** | **Strain*^a^*** | **Isolates from other sourdough showing the same profile** | **Cluster*^b^*** | **Closest relative (% identity)*^c^*** |
| --- | --- | --- | --- | --- |
| C | A3 | None | I | *Lactobacillus curvatus* (99) |
|  | A9 (A10) | None | I | *L. curvatus* (99) |
|  | A11 (A12-14) | None | I | *L. curvatus* (99) |
|  | A5 (A6) | None | I | *L. curvatus* (99) |
|  | A7 | None | I | *L. curvatus* (99) |
|  | A4 | None | I | *L. curvatus* (99) |
|  | A8 | None | I | *L. curvatus* (99) |
|  | B1 | None | II | *L. curvatus* (99) |
|  | B8 (B11, B13) | None | II | *L. curvatus* (99) |
|  | B5 | None | II | *Pediococcus pentosaceus* (100) |
|  | B2 (B4) | None | II | *L. curvatus* (99) |
|  | B9 (B12, B14, B15) | None | II | *L. curvatus* (99) |
|  | B6 (B7) | None | II | *P. pentosaceus* (100) |
| C-IF | A1 (A2-12) | None | UC | *P. pentosaceus* (100) |
|  | B1 (B2-12) | None | VI | *P. pentosaceus* (100) |
| D1-IF | A1 (A2-6, A8-12) | D2-IF A11-12, D2-IF A14-15; D7-IF B2-6, D7-IF B10-15 | IV | *P. pentosaceus* (100) |
|  | A7 (A13, A15) | None | IV | *P. pentosaceus* (100) |
|  | A14 | D3-IF A1, D3-IF A3-14; D7-IF B7-9; D8-IF B1-15 | IV | *P. pentosaceus* (100) |
|  | B1 (B2-14) | None | V | *P. pentosaceus* (100) |
|  | B15 | None | V | *P. pentosaceus* (100) |
| D2-IF | A1 (A2-10, A13) | None | IV | *P. pentosaceus* (100) |
|  | B1 (B2-15) | None | IV | *P. pentosaceus* (100) |
| D3-IF | A2 | None | IV | *P. pentosaceus* (100) |
|  | B1 (B2-3, B5-15) | None | IV | *P. pentosaceus* (100) |
| D4-IF | A1 (A2-14) | None | III | *P. pentosaceus* (100) |
|  | A15 | None | III | *P. pentosaceus* (100) |
|  | B1 (B2-7, B9, B11-15) | None | III | *P. pentosaceus* (100) |
|  | B8 | None | III | *L. curvatus* (99) |
| D5-IF | A1 (A2-5, A7-10, A12-15) | None | III | *P. pentosaceus* (100) |
|  | B1 | None | IV | *P. pentosaceus* (100) |
|  | B2 (B3-15) | None | IV | *P. pentosaceus* (100) |
| D6-IF | A1 (A2-15; B1-4) | None | IV | *P. pentosaceus* (100) |
|  | B5 | None | IV | *P. pentosaceus* (100) |
|  | B6 (B7-15) | None | IV | *P. pentosaceus* (100) |
| D7-IF | B1 | None | IV | *P. pentosaceus* (100) |
|  | A1 (A2-3, A9-15) | None | IV | *P. pentosaceus* (100) |
| D8-IF | A1 (A2-15) | None | UC | *P. pentosaceus* (100) |

***^a^*** Isolate(s) in brackets showed the same RAPD profiles as the identified strain.

***^b^*** RAPD-PCR cluster. Clusters are numbered with Roman numerals from I to VI; UC, unclustered.

***^c^*** Species showing the highest identity (%) to the strain isolated from sourdough. The percentage of identity was that shown by performing multiple sequence alignments in BLAST.
