# Supplementary material for: Different Flour Microbial Communities Drive to Sourdoughs Characterized by Diverse Bacterial Strains and Free Amino Acid Profiles
Source: Front Microbiol. 2016 Nov 8;7:1770. doi: 10.3389/fmicb.2016.01770 (PMC5099235; doi:10.3389/fmicb.2016.01770)
Supplement: Supplementary file 6 [file Table6.DOC]

Supplementary Material

**Different flour microbial communities drive to sourdoughs characterized by diverse bacterial strains and free amino acid profiles**

**Giuseppe Celano, Maria De Angelis, Fabio Minervini*, Marco Gobbetti**

*** Correspondence:** Corresponding Author: [fabio.minervini@uniba.it](mailto:fabio.minervini@uniba.it)

**TABLE S6.** Strains of *Saccharomyces cerevisiae* isolated from the mature sourdoughs prepared with irradiated durum wheat flour (IF).

| **Strain*^a^*** | **Cluster*^b^*** |
| --- | --- |
| D2-IF 1  D4-IF 8 (D4-IF 9; D6-IF 5)  D2-IF 2 | I  I  I |
| D6-IF 3 | I |
| D7-IF 1 (D7-IF 2; D2-IF 5, 7-8, 10; D4-IF 1; D6-IF 6-7, 9) | UC |
| D2-IF 3 | II |
| D2-IF 6 (D2-IF 9) | II |
| D4-IF 4 (D4-IF 6-7; D2-IF 4) | II |
| D4-IF 5 | II |
| D8-IF 10 (D8-IF1-9; D4-IF 2-3, 10; D6-IF 1-2, 4, 8, 10; D7-IF 4-5, 8, 10) | UC |
| D7-IF7 | UC |

*^a^* Isolate(s) in brackets showed the same RAPD profiles as the identified strain.

*^b^* RAPD-PCR cluster. Clusters are numbered with Roman numerals from I to II; UC, unclustered.
