# Supplementary material for: Different Flour Microbial Communities Drive to Sourdoughs Characterized by Diverse Bacterial Strains and Free Amino Acid Profiles
Source: Front Microbiol. 2016 Nov 8;7:1770. doi: 10.3389/fmicb.2016.01770 (PMC5099235; doi:10.3389/fmicb.2016.01770)
Supplement: Supplementary file 7 [file Table7.DOC]

Supplementary Material

**Different flour microbial communities drive to sourdoughs characterized by diverse bacterial strains and free amino acid profiles**

**Giuseppe Celano, Maria De Angelis, Fabio Minervini*, Marco Gobbetti**

*** Correspondence:** Corresponding Author: fabio.minervini@uniba.it

**TABLE S7** Alpha diversity indexes of *Bacteria* (16S rRNA) found in the sourdoughs prepared with irradiated durum wheat flour (IF) or non-irradiated flour (C), and in non-inoculated doughs, after the first fermentation, prepared with irradiated (C-IF-1^st^) or non-irradiated (C-1^st^) flour.

| Sample | Number of OTU | Chao1 richness | Shannon diversity index |
| --- | --- | --- | --- |
| D1-IF | 16 | 15 | 1.90 |
| D2-IF | 9 | 9 | 1.49 |
| D3-IF | 10 | 10 | 1.31 |
| D4-IF | 6 | 7 | 1.31 |
| D5-IF | 8 | 8 | 1.34 |
| D6-IF | 6 | 6 | 1.22 |
| D7-IF | 10 | 10 | 1.45 |
| D8-IF | 8 | 7 | 1.27 |
| C-IF-1^st^ *^a^* | 54 | 54 | 3.29 |
| C-IF *^b^* | 11 | 11 | 1.56 |
| C-1^st^ *^c^* | 26 | 27 | 2.56 |
| C *^d^* | 22 | 22 | 2.18 |

*^a^* Dough prepared with irradiated flour, without inoculation and analyzed at the end of the first fermentation

*^b^* Dough prepared with irradiated flour, without inoculation and analyzed at the end of the fifth back-slopping

*^c^* Dough prepared with non-irradiated flour and analyzed at the end of the first fermentation

*^d^* Dough prepared with non-irradiated flour and analyzed at the end of the fifth back-slopping
