# Supplementary material for: Different Flour Microbial Communities Drive to Sourdoughs Characterized by Diverse Bacterial Strains and Free Amino Acid Profiles
Source: Front Microbiol. 2016 Nov 8;7:1770. doi: 10.3389/fmicb.2016.01770 (PMC5099235; doi:10.3389/fmicb.2016.01770)
Supplement: Supplementary file 8 [file Presentation1.PPTX]

## Slide 1
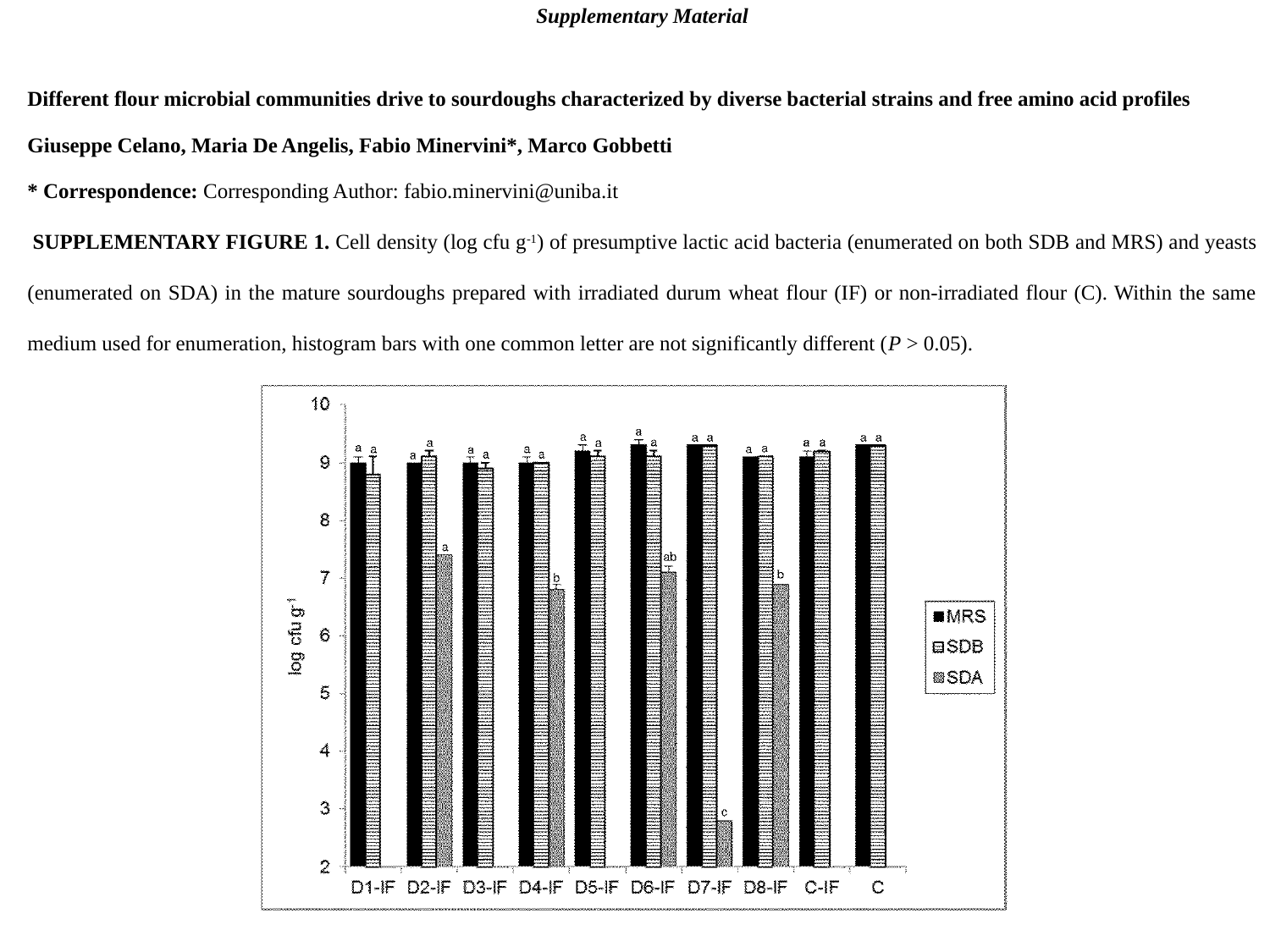

Supplementary Material
Different flour microbial communities drive to sourdoughs characterized by diverse bacterial strains and free amino acid profiles
Giuseppe Celano, Maria De Angelis, Fabio Minervini*, Marco Gobbetti
* Correspondence: Corresponding Author: fabio.minervini@uniba.it
 SUPPLEMENTARY FIGURE 1. Cell density (log cfu g-1) of presumptive lactic acid bacteria (enumerated on both SDB and MRS) and yeasts (enumerated on SDA) in the mature sourdoughs prepared with irradiated durum wheat flour (IF) or non-irradiated flour (C). Within the same medium used for enumeration, histogram bars with one common letter are not significantly different (P > 0.05).
